# Molluscan mega-hemocyanin: an ancient oxygen carrier tuned by a ~550 kDa polypeptide

**DOI:** 10.1186/1742-9994-7-14

**Published:** 2010-05-13

**Authors:** Bernhard Lieb, Wolfgang Gebauer, Christos Gatsogiannis, Frank Depoix, Nadja Hellmann, Myroslaw G Harasewych, Ellen E Strong, Jürgen Markl

**Affiliations:** 1 Institute of Zoology, Johannes Gutenberg University, 55099 Mainz, Germany; 2 Institute of Molecular Biophysics, Johannes Gutenberg University, 55099 Mainz, Germany; 3 Department of Invertebrate Zoology, Smithsonian Institution, National Museum of Natural History, MRC, 163, PO Box 37012, Washington, DC 20013-7012, USA

## Abstract

**Background:**

The allosteric respiratory protein hemocyanin occurs in gastropods as tubular di-, tri- and multimers of a 35 × 18 nm, ring-like decamer with a collar complex at one opening. The decamer comprises five subunit dimers. The subunit, a 400 kDa polypeptide, is a concatenation of eight paralogous functional units. Their exact topology within the quaternary structure has recently been solved by 3D electron microscopy, providing a molecular model of an entire didecamer (two conjoined decamers). Here we study keyhole limpet hemocyanin (KLH2) tridecamers to unravel the exact association mode of the third decamer. Moreover, we introduce and describe a more complex type of hemocyanin tridecamer discovered in fresh/brackish-water cerithioid snails (*Leptoxis*, *Melanoides*, *Terebralia*).

**Results:**

The "typical" KLH2 tridecamer is partially hollow, whereas the cerithioid tridecamer is almost completely filled with material; it was therefore termed "mega-hemocyanin". In both types, the staggering angle between adjoining decamers is 36°. The cerithioid tridecamer comprises two typical decamers based on the canonical 400 kDa subunit, flanking a central "mega-decamer" composed of ten unique ~550 kDa subunits. The additional ~150 kDa per subunit substantially enlarge the internal collar complex. Preliminary oxygen binding measurements indicate a moderate hemocyanin oxygen affinity in *Leptoxis *(p50 ~9 mmHg), and a very high affinity in *Melanoides *(~3 mmHg) and *Terebralia *(~2 mmHg). Species-specific and individual variation in the proportions of the two subunit types was also observed, leading to differences in the oligomeric states found in the hemolymph.

**Conclusions:**

In cerithioid hemocyanin tridecamers ("mega-hemocyanin") the collar complex of the central decamer is substantially enlarged and modified. The preliminary O_2 _binding curves indicate that there are species-specific functional differences in the cerithioid mega-hemocyanins which might reflect different physiological tolerances of these gill-breathing animals. The observed differential expression of the two subunit types of mega-hemocyanin might allow individual respiratory acclimatization. We hypothesize that mega-hemocyanin is a key character supporting the adaptive radiation and invasive capacity of cerithioid snails.

## Background

**Figure 1:**
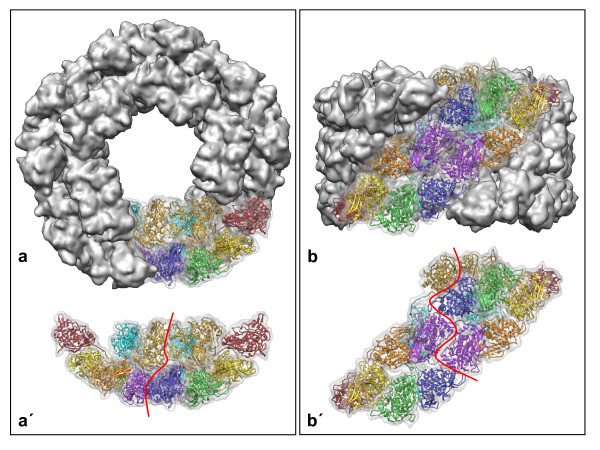
**Typical gastropod hemocyanin decamer (a, b), and extracted subunit dimer (a', b')**. In **a **and **b**, one out of five subunit dimers is transparent to reveal the underlying molecular model of KLH1 published recently [2]. The 3D volume shown here in grey was calculated from this molecular model at 9-Å resolution. Red lines in **a' **and **b' **mark the borderline between the two constituent 400 kDa subunits, each encompassing eight different functional units (red, FU-a; yellow, FU-b; green, FU-c; orange, FU-d; purple, FU-e; blue, FU-f; cyan, FU-g; gold, FU-h). Note that FU-a to FU-f form the cylinder wall, whereas FU-g and FU-h constitute the collar complex. Although the two subunits are identical in primary structure, they are folded into two different conformations to form an asymmetric homo-dimer [1].

The most urgent physiological need of animals is a constant supply of oxygen, usually provided by allosteric respiratory proteins such as the red, iron-based protein hemoglobin. In most molluscs, this crucial role is played by the blue, copper-containing protein hemocyanin. Its basic hemocyanin oligomer, the decamer, is composed of five identical subunit dimers forming a cylinder with a collar complex at one end [1,2] (Figure 1). Gastropods express a didecamer assembled from two conjoined decamers, with the collar complexes facing outward at the ends. The subunit, a ~400 kDa polypeptide, is a concatenation of eight paralogous functional units termed FU-a to FU-h, each with a single copper active site for reversible oxygen binding [3]. The exact topology of the functional units within the didecamer has only recently been established through 3D electron microscopy of keyhole limpet hemocyanin isoform 1 [2]. Keyhole limpet hemocyanin (KLH) is an established immune response modifier and hapten carrier [4,5]. The two KLH isoforms (termed KLH1 and KLH2) are homo-oligomers, each assembled from a single subunit type of ~400 kDa. KLH1 forms didecamers and didecamer clusters, whereas KLH2 forms didecamers and multidecamers. The two subunit types differ substantially in primary structure and most probably furcated by a gene duplication event some 340 million years ago (for literature, see [2-5]).

**Figure 2:**
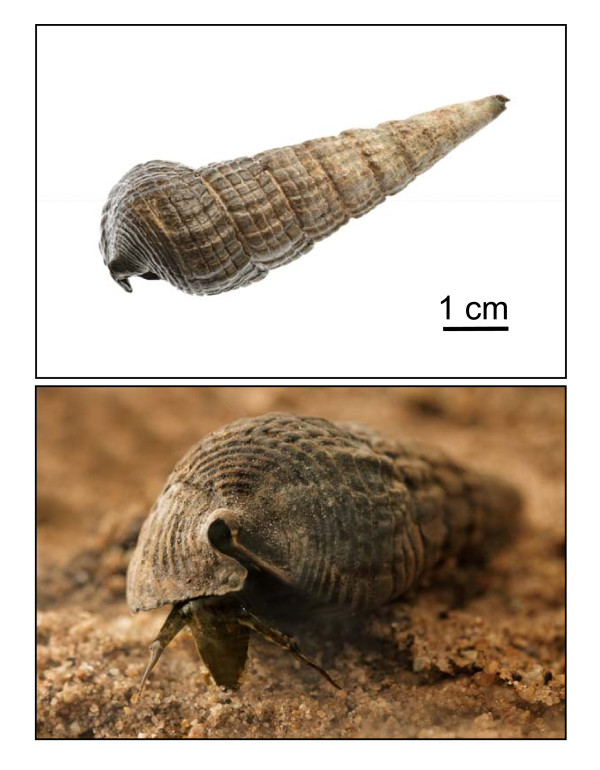
***Terebralia palustris *(Cerithioidea), from the mangrove mud near Gazi, Kenya**. Images taken by Christoph Kühne.

The function of gastropod hemocyanins is characterized by moderate cooperativity, Bohr effect and oxygen affinity; high oxygen affinities as required under hypoxic conditions are not observed [6]. In warm hypoxic ponds this might limit the diving time of lung breathers such as *Lymnea *[7]. The lung breathing planorbid snails (e.g. *Biomphalaria*) evolved an intriguing respiratory alternative for prolonged diving, namely a multimeric hemoglobin with high oxygen affinity [7,8]. The convergent occurrence of hemoglobins in some bivalves that thrive under hypoxic conditions [see references in [8]] also suggests that molluscan hemocyanin has been unable to evolve into high affinity forms, needed especially by gill breathers for survival under hypoxic conditions. We have now found in the Cerithioidea a unique complex "mega-hemocyanin" tridecamer which is probably capable of adapting to a much broader range of oxygen affinities than typical hemocyanin. Cerithioid snails (Figure 2) span a broad range of aquatic habitats, where they are often important components of the respective communities [9,10]. The present study provides the first structural and a preliminary functional analysis of this newly detected hemocyanin type.

Typical gastropod hemocyanin didecamers tend to bind additional decamers at one or both ends, thereby forming tubular tridecamers and larger multidecamers; a well-studied example is KLH2 [4]. However, the exact association mode of the additional decamers remained unknown and therefore was elucidated in the present context, in order to better understand the unique structure of the novel mega-hemocyanin tridecamer.

## Results and Discussion

### Architecture of the KLH2 tridecamer

**Figure 3:**
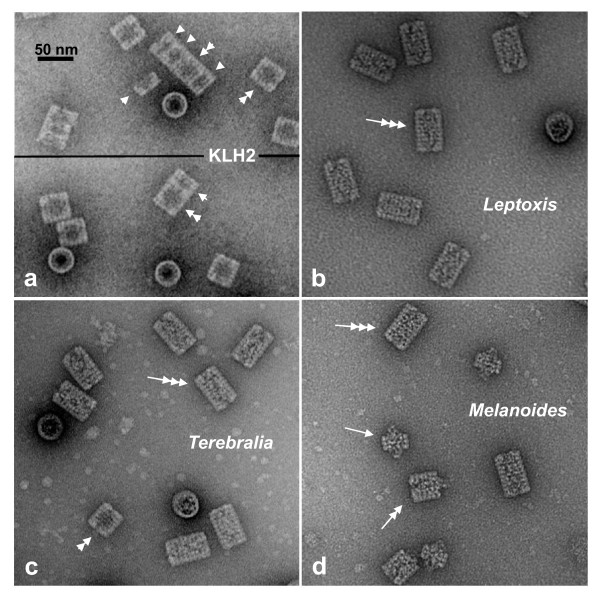
**Transmission electron microscope images of negatively stained gastropod hemocyanins**. (**a**) KLH2 (from *Megathura crenulata*), showing typical didecamers (double arrowhead), typical tridecamers build from a decamer (arrowhead) and a didecamer (double arrowhead), and a larger multidecamer. (**b**) *Leptoxis carinata *hemocyanin; note exclusive presence of mega-tridecamers (triple arrow). (**c**) *Terebralia palustris *hemocyanin, showing mostly mega-tridecamers (triple arrow), but also typical didecamers (double arrowhead). (**d**) *Melanoides tuberculata *hemocyanin, showing a mixture of mega-decamers (arrow), mega-didecamers (double arrow) and mega-tridecamers (triple arrow).

**Figure 4:**
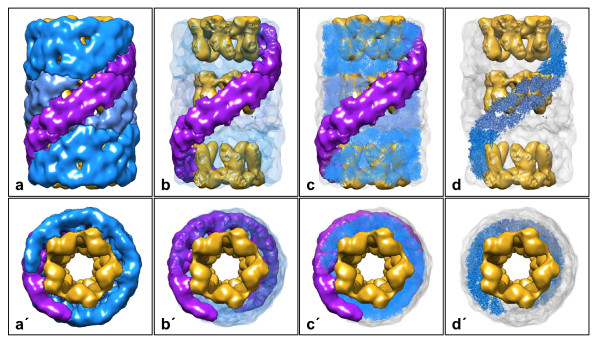
**The KLH2 tridecamer at 30-Å resolution**. (**a**) Side view and (**a'**) top view of the 3D reconstruction at 30-Å resolution as obtained from negatively stained EM images. Segmentation was based on the fitting of the published molecular model of KLH1 [2]. Fenestration and right-handed helical appearance of the cylinder wall results from the oblique arrangement of subunit dimers (purple). Note that this helix is steadily continued in the additional (the upper) decamer. (**b**, **b'**) The same situation as in **a**, **a'**, but with most of the cylinder wall transparent to reveal the collar complexes (golden). (**c**, **c'**) Three copies of the molecular model of the KLH1 decamer (for the latter, see [2]) fitted into the 30-Å structure, and shown with the collar complexes and three consecutive subunit dimers as extracted from the 30-Å structure. Note that the 3D volume is somewhat larger than the molecular model; this is due to the negative staining and flattening of the particles used for 3D reconstruction. (**d**, **d'**) Three consecutive subunit dimers of the fitted molecular model (ribbon style). This helical association mode is also continued in the larger multidecamers. Formally, the turn between the didecamer and the attached decamer is 36°, but it is actually 3 × 36° = 108° if topologically identical positions are compared.

**Figure 5:**
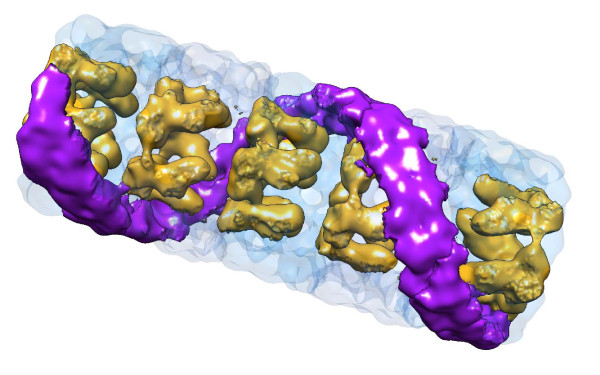
**Hypothetical structure of a KLH2 pentadecamer**. This model was obtained by fusing two extracted decamers, at a rotation angle of 36°, with the 30-Å structure of the KLH2 tridecamer. Orientation of the five decamers is indicated by their collar complexes (golden); note that the right-handed helical structure of the cylinder wall is steadily continued, as shown here in each decamer for a subunit dimer (purple). The pitch of the surface helical feature, with respect to the longitudinal axis, is 45° (see also [2,15]). The pitch height (frequency of twist) of the right-handed helix is 4.5 subunit dimers, meaning that in the pentadecamer the helix completes 360°.

In the electron microscope, purified KLH2 shows the presence of di-, tri- and multidecamers (Figure 3a), which confirms earlier observations [11-15]. We enriched the KLH2 tridecamer fraction by gel filtration chromatography and produced, by electron microscopy of negatively stained molecules and single particle analysis, a 30-Å reconstruction (Figure 4a, a', b, b'). Although this resolution was low, it reproducibly allowed automated rigid-body fitting of three copies of the molecular model of the KLH1 decamer (Figure 4c, c', d, d'); this model was available from a previous study [2]. The results clearly show that within the tridecamer, the additional decamer is rotated 36° with respect to the didecamer. This fits previous electron microscopical observations [15]. Indeed, it can be demonstrated using several plaster 3D models of the KLH1 decamer, obtained by rapid prototyping of the published 9-Å volume [2], that a rotation angle of 36° joins them in the most stable manner. It should be noted that 36° is also the staggering angle of the two constituent decamers within the "nucleating" didecamer of KLH1 [2], although in this case the two decamers are assembled with their "open" ends facing each other, and their closed ends (with the collar complex) facing outwards. In contrast, in tri- and multidecamers the additional decamers are attached with their open end to a closed end (Figure 5; see also [16]).

**Figure 6:**
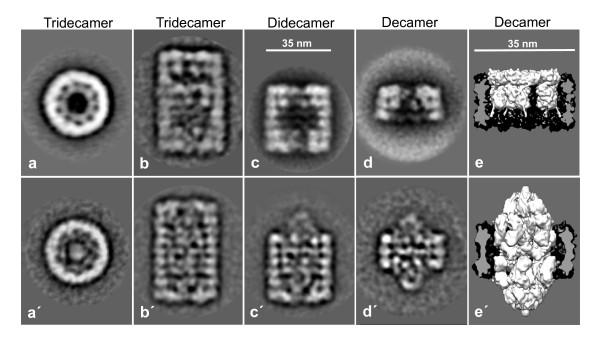
**Different oligomeric states of KLH2 (a-e) and *Leptoxis carinata *hemocyanin (a'-e')**. (**a-d; a'-d'**) Class sum images of negatively stained single particles collected from electron micrographs. (**e**, **e'**) 3D models shown as longitudinal section through the cylinder wall to reveal the fundamentally different collar complexes. Note that the flattening effect is more obvious in KLH2 (upper panel).

Molluscan hemocyanin decamers are assembled from five oblique subunit dimers, resulting in five major and five minor right-handed helical grooves encarving the surface of the cylinder wall. Due to the decamer↔decamer staggering angle of 36°, these grooves are steadily continued in the didecamer (see Figure 4) which electron microscopists observed several decades ago [17,18]. The recent 9-Å 3D structure of KLH1 ultimately confirmed this for the didecamer [2], and the present results show, for the first time, steady continuation of the helical grooves in the tridecamer. From spatial constraints it is clear that this association mode is also true for the additional decamers in the larger KLH2 multidecamers, as illustrated in Figure 5 for a pentadecamer. In Figure 6a-d, representations of the KLH2 tridecamer and smaller oligomers derived from electron microscopical images are shown, together with a 3D model of the basic decamer (Figure 6e).

### Electron microscopy of *Leptoxis carinata *mega-hemocyanin

*Leptoxis carinata *(Pleuroceridae) is a small North American freshwater snail populating rocks in shallow, well-aerated water [19]. During a routine electron microscopic survey of hemocyanin samples from this snail we detected exclusively tridecamers (Figure 3b), which is unprecedented for a molluscan hemocyanin. The typical hemocyanin tridecamer observed in KLH2 (see Figure 3a) and many other molluscs (e.g. [11-13,16]) is a transient state between the comparatively stable didecamers and larger multidecamers; it is therefore generally present as a minor component. In previous careful dissociation/reassembly studies, conditions that would transform the bulk of material into tridecamers were not found [13-15]. Therefore, it was completely unexpected to find here an exclusive population of fully stable tridecamers.

**Figure 7:**
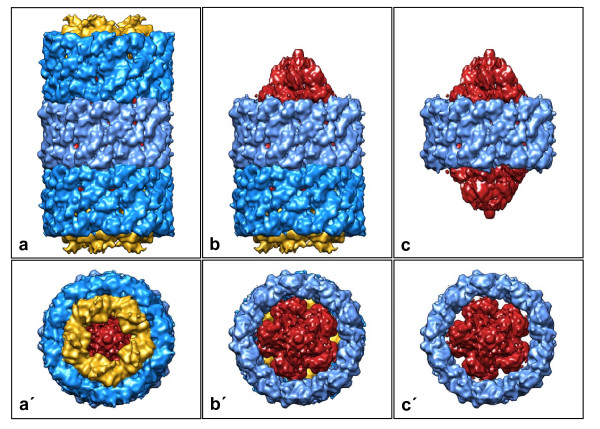
**3D reconstruction of *Leptoxis carinata *mega-hemocyanin at 13-Å resolution**. (**a**, **a'**) Mega-tridecamer, with the three decamers and the collar complexes color-coded (typical decamers: blue/golden; mega-decamer: light-blue/red); (**b**, **b'**) extracted mega-didecamer; (**c**, **c'**) extracted mega-decamer. The peripheral decamers and the wall of the central decamer conform to the molecular model of KLH1 [1]. A detailed model of the central collar complex (red) will require a higher resolution 3D reconstruction and the complete amino acid sequence of the 550 kDa polypeptide.

**Figure 8:**
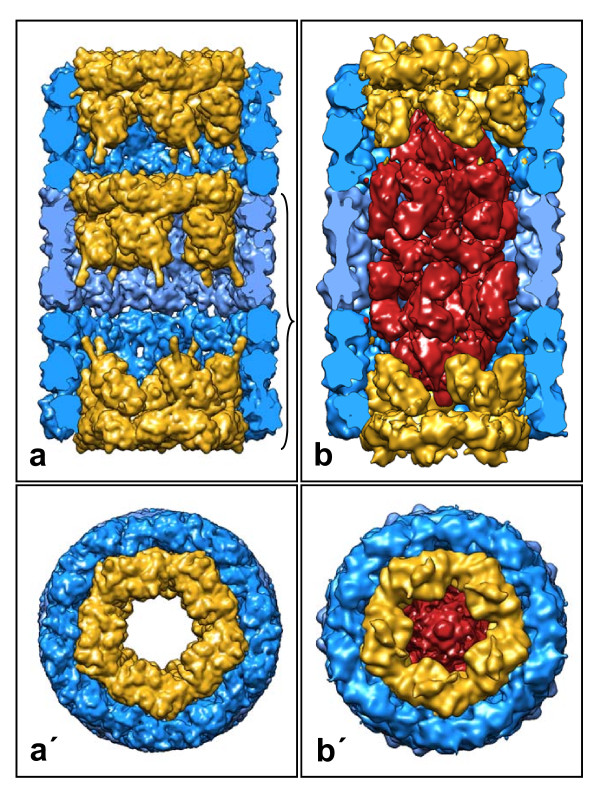
**Model of the KLH2 tridecamer (a, a'), and 13-Å reconstruction of the *Leptoxis carinata *mega-tridecamer (b, b')**. Side views (upper panel) are shown as a longitudinal section through the cylinder wall to reveal the collar complexes. The volumes in **a**, **a' **were calculated, at 9-Å resolution, from 3 copies of the molecular model of the KLH1 decamer [2] that had been fitted into the 30-Å reconstruction of the KLH2 tridecamer (see Figure 4). The bracket in **a **indicates the „nucleating” didecamer.

Moreover, these tridecamers looked abnormal. In contrast to the partially hollow hemocyanin tridecamers of other molluscs (see Figure 6a, b), the centre of these tridecamers is almost completely filled with material (Figure 6a', b'). Partial dissociation of the *Leptoxis *tridecamers induced by prolonged storage revealed two unique sub-structures: a didecamer with a single mass extension (Figure 6c'), and a decamer with two extensions (Figure 6d'). Typical decamers were also detected. 3D electron microscopy of the *Leptoxis *tridecamer under cryo conditions yielded a ~13-Å reconstruction (Figure 7). It revealed that this "mega-hemocyanin" is actually composed of two different units: the flanking typical decamer, with its collar complex shifted towards one cylinder opening (point-group C5 symmetry, see Figure 6e), and the central "mega-decamer" with a much larger and more symmetrical collar complex (probably point-group D5 symmetry; Figure 6e'). The difference between a typical hemocyanin tridecamer and mega-hemocyanin is illustrated in Figure 8.

In the typical gastropod hemocyanin collar complex, five FU-g pairs form five hidden "arcs" protruding into the cylinder lumen. Moreover, five FU-h pairs build five exposed "slabs"; the latter are arranged as a flat ring (Figure 8a, a'). In spite of the fully anti-parallel subunit arrangement in the wall, the whole collar complex is shifted towards one end of the cylinder (see Figure 1). This has been explained on the basis of the subunits forming an asymmetric homo-dimer [2], and it is amazing that in mega-hemocyanin this apparently has turned into a more symmetric homo-dimer (Figure 8b, b'). However, such a mutation is not unique, but has a parallel in cephalopod hemocyanins. The latter always occur as single decamers, with FU-h missing [20,21]. In *Nautilus *hemocyanin, the wall architecture of this decamer and the asymmetric position of FU-g pairs forming the collar are indistinguishable from the situation in KLH1 [1,2,22]. However, in *Sepia *hemocyanin an additional FU, resulting from gene duplication of FU-d, also contributes to the collar complex by substantially enlarging it and forcing it into a more symmetrical structure [23].

Mutations of the collar complexes in cerithioid and sepioid hemocyanins clearly result from convergent evolution, but they demonstrate that such rearrangements of the collar are possible without destabilizing the hemocyanin particle in the intermediate phases. This is due to inherent stability of the isolated wall which has been demonstrated in cases in which the collar complex is lacking [8].

### Subunit composition of mega-hemocyanin

**Figure 9:**
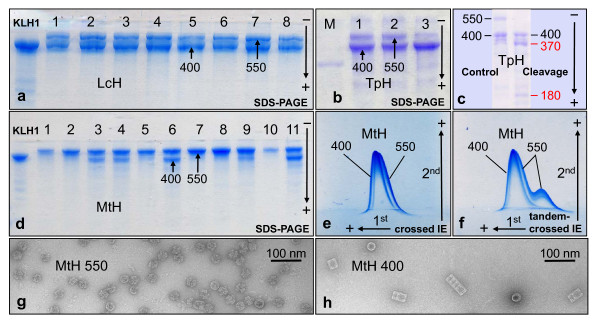
**Subunit structure and reassembly of mega-hemocyanin**. (**a**) SDS-PAGE of *Leptoxis carinata *hemocyanin (LcH); note the constant ratio of the two subunits in the different individuals. (**b**) SDS-PAGE of whole *Terebralia palustris *hemocyanin (TpH, sample 1), and of gel filtration chromatography fractions enriched in mega-tridecamers (sample 2) and typical didecamers (sample 3), respectively. M, myosin marker (205 kDa) (**c**) SDS-PAGE of intact *Terebralia *hemocyanin ("control", left lane) and of *Terebralia *hemocyanin after limited tryptic cleavage for 3 hours ("cleavage", right lane); note occurrence of two subunit fragments derived from the 550 kDa subunit. (**d**) SDS-PAGE of *Melanoides tuberculata *hemocyanin (MtH); note variable ratio of the two subunits in the different individuals. (**e**) Crossed immunoelectrophoresis (IE) of *Melanoides tuberculata *hemocyanin (MtH, sample 6 in **d**), showing that the two subunits are immunologically distinct. (**f**) Tandem-crossed immunoelectrophoresis (samples 6 and 7 in **d**) to identify both peaks. (**g**, **h**) Reassembly experiments with the individual MtH subunits; note exclusive formation of mega-decamers in **g**, and of typical hemocyanin oligomers in **h**.

**Figure 10:**
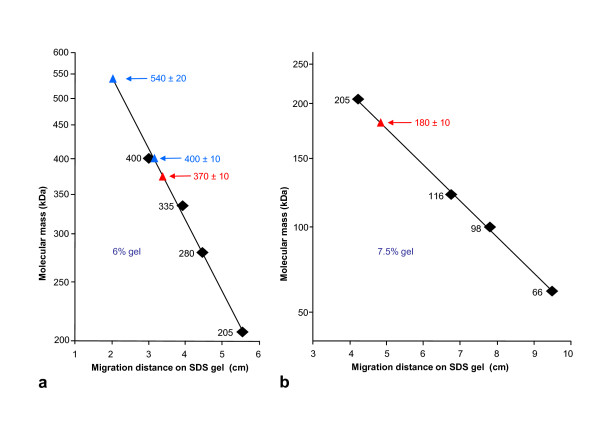
**Molecular mass determination of *Terebralia *hemocyanin by SDS-PAGE**. (**a**) Apparent molecular mass of both intact subunit types (blue triangles), and of the larger tryptic fragment (red triangle) obtained from the larger subunit. As molecular mass markers we used KLH1 (400 kDa [2]), *Nautilus pompilius *hemocyanin (335 kDa [21]), *Rapana thomasiana *hemocyanin (400 kDa and 280 kDa natural fragment [45]), and myosin (205 kDa, standard marker). Note that the 540 kDa value is obtained by graphical extrapolation. (**b**) Apparent molecular mass of the smaller tryptic fragment (red triangle) obtained from the larger subunit; a standard mixture of molecular mass markers (myosin, β-galactosidase, phosphorylase A, bovine serum albumin) was applied. Note that the two fragments summate to 550 kDa.

In addition to the typical ~400 kDa subunit, SDS-PAGE of *Leptoxis carinata *hemocyanin shows a previously unknown, significantly larger polypeptide (Figure 9a). A comparable pattern was obtained from *Leptoxis praerosa *hemocyanin (not shown) and *Terebralia palustris *hemocyanin (Figure 9b). *Terebralia palustris *(Potamididae) is a mangrove snail from Kenya. Biophysical molecular mass determination of molluscan hemocyanin subunits is generally difficult due to the enormous size of the subunits and the lack of marker proteins in this range, and therefore a matter of debate (see [1,6]). In the past we applied a variety of methods [12,13] and ultimately found, as soon as "true" molecular masses predicted from primary structures became available, that at least in our hands SDS-PAGE yielded the most accurate results (e.g. [3,21]). Molluscan hemocyanins are glycosylated, but their glycan content is generally not high enough to cause irregular migration on SDS gels. For example, in case of *Haliotis tuberculata *hemocyanin, ~400 kDa was determined by SDS-PAGE, and 392 kDa was later predicted from the amino acid sequence; from their total yield, the glycans might well account for the balance of *ca. *8 kDa [3]. From these previous studies we had available various well-characterized molluscan hemocyanin subunits and subunit fragments that could be used in SDS-PAGE as molecular mass markers of appropriate size. Using KLH1 (400 kDa, see Figure 9a) as the largest marker, we determined ~540 kDa for the larger mega-hemocyanin subunit; however, critically, the larger subunit was determined by graphical extrapolation (Figure 10a).

In previous studies on the typical molluscan hemocyanin subunits, limited proteolytic cleavage (by using low amounts of proteases) proved to be a strong tool in analyzing the functional unit concatenation. Under the appropriate conditions, certain proteases cleave one or several of the exposed, elongated linkers between functional units, whereas the globular core of the functional units remains intact. In SDS-PAGE this yields well-defined subunit fragments containing one, two or several functional units (e.g. [12,13]). Using this approach on *Terebralia *hemocyanin, we obtained a very complex cleavage pattern when exposing the subunit mixture to 5% trypsin (not shown). However, if 5% trypsin was applied to the intact oligomers, after subsequent denaturation a clear pattern was revealed in SDS-PAGE: The 400 kDa subunit was still intact, but the larger subunit yielded two well defined cleavage products (Figure 9c), with 370 ± 10 and 180 ± 10 kDa, respectively (Figure 10a, b); the process was still incomplete after 1 hour but complete after 3 hours. Together, the two fragments represent ~550 kDa, which is taken here for the apparent molecular mass of the intact subunit, as we still lack the amino acid sequence. The stability of the typical hemocyanin decamers of *Terebralia *to this protease treatment conforms with our previous observations on keyhole limpet and *Haliotis *hemocyanin. In contrast, the ~550 kDa subunit apparently has a tryptic cleavage site that is freely accessible for the enzyme even in the native quaternary structure.

In all *Leptoxis *individuals studied thus far (n = 51), the ratio of the 400 kDa to 550 kDa subunit is *ca. *2:1 (see Figure 9a), suggesting that this is indeed the correct ratio within the mega-tridecamer. Interestingly, in the studied *Terebralia *individuals (n = 13), the ratio of the 400 kDa subunit was higher than in *Leptoxis*, and was further increased in samples enriched in didecamers *via *gel filtration chromatography (see Figure 9b). The electron microscope showed typical didecamers in addition to mega-tridecamers (Figure 3c). In *Melanoides tuberculata *(Thiaridae), variable proportions of the two subunits are present, but in all studied individuals (n = 22) with a clear excess of the 550 kDa subunit (Figure 9d). Electron microscopy revealed a variable mixture of mega-hemocyanin oligomers (Figure 3d). Some individuals even lack the 400 kDa subunit and possess mega-decamers exclusively in their hemolymph. The two subunit types are immunologically distinct (Figure 9e, f), which usually corresponds to major differences (>30%) in primary structure. Reassembly experiments with isolated *Melanoides *subunits confirmed that the 550 kDa subunit exclusively forms mega-decamers (Figure 9g), whereas the 400 kDa subunit produces typical decamers which further assemble into typical di-, tri- and multidecamers (Figure 9h).

The presence of two or more different subunit types within the same hemocyanin particle is usual in arthropods [24,25], but rarely observed in molluscs. In the latter, hemocyanin heterogeneity is obvious at the level of the functional units [3,12,13,20]. In keyhole limpet hemocyanin and *Haliotis tuberculata *hemocyanin, it has been demonstrated by many reassembly experiments that the two 400 kDa subunit isoforms are unable to co-polymerize into hetero-oligomers: reassembly attempts from subunit mixtures always resulted in distinct homo-oligomers which fits the observation that the native hemocyanin is devoid of hetero-oligomers [14,26]. The only previously published example of a mollusc with hetero-oligomeric hemocyanin is *Concholepas*, but this animal has a typical hemocyanin decamer made from two types of 400 kDa subunit [27]. A situation such as we have now detected in cerithioid snails has not previously been reported.

### Oxygen binding of mega-hemocyanin

In contrast to other gastropod hemocyanins [6], oxygen binding curves of whole hemocyanin from *Leptoxis praerosa*, *Melanoides *and *Terebralia *all indicate low cooperativity (Hill coefficient of 1.0-1.2 at half-saturation). Oxygen affinity (p50) is moderate in *Leptoxis *(mega-tridecamers: 9 ± 1 mmHg at pH 7.6; 1 mmHg ~133 Pa), but very high in *Melanoides *(mega-decamers: 2.6 ± 0.5 mmHg at pH 7.6) and even higher in *Terebralia *(mostly mega-tridecamers and some typical didecamers: 1.8 ± 0.5 mmHg at pH 7.6). These data are preliminary, but seem to reflect the different physiological tolerances of these gill-breathing species: *Leptoxis *is stenotrophic and requires swift-flowing, well-oxygenated waters; in contrast, *Melanoides *is found in quiet waters and can tolerate stagnant, eutrophic conditions, and *Terebralia *thrives in warm mangrove mud. In these cases, the observed difference in p50 indicates an evolutionary adaptation, and could mean the difference between survival or death.

Planorbid freshwater snails such as *Biomphalaria glabrata *are lung breathers, adapted to prolonged diving in warm ponds; they switched from "blue to red blood" by almost exclusively expressing a multimeric hemoglobin with p50 ~6 mmHg [7,8]. It has been suggested that hemocyanin is probably unable to evolve into high affinity oxygen-binding forms needed under conditions of hypoxia [28]. However, mega-hemocyanin seems to have this capacity and possibly has the added advantage of allowing respiratory acclimatization through differential expression of the 400 kDa and 550 kDa subunit types. It also seems to us that the functional plasticity of mega-hemocyanin, notably its ability to function properly under hypoxic as well as normoxic conditions, exceeds that of typical hemocyanin and also of multimeric hemoglobin.

## Conclusions

Our previous 9-Å structure of the KLH1 didecamer essentially solved the overall architecture, intricate subunit pathway and near-atomic structure of a gastropod hemocyanin decamer and didecamer [2]. The present 3D model of the KLH2 tridecamer (see Figure 4) and its extrapolation to larger KLH2 multidecamers (see Figure 5) shows that within all types of KLH molecule, the constituent decamers are rotated 36° with respect to their adjoining decamers, thereby yielding a steady continuation of the right-handed helical grooves characteristic of the cylinder outer wall. As deduced from multiple sequence alignments and biochemical analyses [21,29,30], the different association forms of KLH can serve as structural models for gastropod (and bivalve) typical hemocyanin in general.

The newly detected mega-hemocyanin, apparently a synapomorphy of the Cerithioidea, can be interpreted on the basis of its present 13-Å cryoEM structure and our detailed knowledge on KLH. We provide strong evidence that the mega-tridecamer is assembled from two peripheral typical decamers (each with ten copies of the 400 kDa subunit), and a central mega-decamer made up from ten 550 kDa subunits. The wall of the mega-decamer is apparently constructed according to the typical scheme, which means that the six wall functional units FU-a to FU-f that exist in the 400 kDa subunit are also present in the 550 kDa subunit.

In contrast, the collar complex of the mega-decamer remains unresolved (shown in red in Figures 7 and 8). It is formed by ten copies of the remaining segment of the 550 kDa subunit, encompassing *ca. *250 kDa, respectively *ca. *2000 amino acids. This might correspond to four or five functional units, instead of only two (FU-g and FU-h) in the collar complex of typical hemocyanin. In total, this newly defined collar complex has a molecular mass of 2.5 Megadalton. Whether or not it shows a true D5 symmetry and how the 250 kDa segment is folded requires its full-length sequence and a higher resolution 3D reconstruction.

In view of the wealth of comparative data available on molluscan hemocyanins (for literature, see [6]), the discovery of a substantially modified type such as mega-hemocyanin, and moreover in some of the most abundant snails, was completely unexpected. The 400 kDa subunit has existed for ~740 million years [31] and is wide-spread among the Mollusca [30,32]. The 550 kDa subunit with its isolated occurrence, probably only in the superfamily Cerithioidea [9] which appeared in the Paleozoic, is a more recent evolutionary specialization. The unique 550 kDa hemocyanin subunit is one of the largest polypeptides ever reported, second only to the myoelastic titin family [33].

Cerithioideans are important members of many freshwater communities spanning a broad range of habitats [9], and might be highly suitable for providing fundamental insights into the mechanisms that generate biodiversity, pattern in historical biogeography, and the underlying processes of speciation and radiation [34,35]. We hypothesize that mega-hemocyanin may have been a key character in their success. From our preliminary O_2_-binding data it appears that *Terebralia *and *Melanoides *hemocyanin have unusually high oxygen affinities. If this holds true in future more detailed analyses, mega-hemocyanin might allow *Terebralia *to populate hypoxic mangrove muds, and presumably has contributed to the invasive capacity of *Melanoides *which thrives in disturbed habitats and has been introduced worldwide through the aquarium trade.

## Methods

### Animals, hemocyanin preparation

*Leptoxis carinata *and *Leptoxis praerosa *(Gastropoda, Pleuroceridae) samples were collected in shallow water on rocks at a single location in the Potomac River, Maryland, USA, and at a single location in Cypress Creek, Alabama, USA, respectively. Both species were collected in spring 2009, *L. carinata *additionally in November 2008 and summer 2009. *Melanoides tuberculata *samples were taken throughout 2009 from two independently established freshwater aquaria in the Institute of Zoology, Mainz, Germany. *Terebralia palustris *was collected in summer 2009 in a mangrove mud near Gazi, Kenya. The keyhole limpet *Megathura crenulata*, source of KLH, was kindly provided by the *biosyn *company, Fellbach, Germany. Hemolymph from the following numbers of snails has been studied individually: *L. carinata*: 51 (SDS-PAGE), 12 (EM); *T. palustris*: 13 (SDS-PAGE), 8 (EM); 3 (O_2 _binding); *M. tuberculata*: 22 (SDS-PAGE), 10 (EM), 3 (O_2 _binding). *L. praerosa*: 5 (SDS-PAGE), 3 (EM), 1 (O_2 _binding). In addition, we studied pooled hemocyanin from 4 *L. praerosa *individuals in all three categories.

Generally, the living animals were transported to the laboratory in Mainz for bleeding, and the hemolymph further processed immediately after bleeding. Some individuals were bled immediately after appearing in our laboratory, others were kept in aquaria and bled days or weeks later. Hemolymph was withdrawn by puncturing the foot muscle by a syringe. The following procedures were as described previously [12,13]: Blood cells were removed by centrifugation and the hemocyanin was purified from the supernatant by pelleting it in a Beckman airfuge. The initial hemocyanin concentration in the animals was 10-25 mg/ml. Purified hemocyanin was routinely stored at 4°C in "stabilizing buffer" (50 mM Tris/HCl, pH 7.4, 150 mM NaCl, 5 mM CaCl_2_, 5 mM MgCl_2_). Pure KLH2 was obtained from an animal that lacked KLH1. Different hemocyanin oligomers were separated by gel filtration chromatography using BioGel A15m. The individual *Melanoides *hemocyanin subunits were separated by cutting the respective protein bands from the native PAGE gel, followed by elution. Alternatively, the 550 kDa subunit was obtained from animals that lacked the 400 kDa component. Hemocyanin oligomers were dissociated into native subunits by dialysis overnight against 0.1 M glycine/NaOH buffer of pH 9.6. Reassembly experiments were performed by overnight dialysis of subunits *versus *"stabilizing buffer", containing 100 mM CaCl_2 _and 100 mM MgCl_2_.

### Electrophoresis and limited tryptic cleavage

SDS polyacrylamide gel electrophoresis (SDS-PAGE) was routinely performed with 7.5% acrylamide in the system of Laemmli [36] as described [12,13]. For limited tryptic cleavage, native *Terebralia *hemocyanin (see Figure 3c) dissolved in stabilizing buffer at a final protein concentration of 16 mg/ml was treated for 1, 2, and 3 hours at 37°C with 5% trypsin (Sigma, Taufkirchen, Germany), and then immediately SDS-denatured to inactivate the protease. The resulting fragments were subsequently analysed by SDS-PAGE. The molecular mass of mega-hemocyanin subunits and fragments was graphically determined according to their migration distances in SDS-PAGE, using several molluscan hemocyanins and a commercial protein standard mixture as markers (for details, see Figure 10); 5%, 6% and 7.5% acrylamide concentrations were applied in this case. Crossed and tandem-crossed immunoelectrophoresis with the system of Weeke [37] was performed as described [12,13], using rabbit anti-*Melanoides *hemocyanin antibodies for the second dimension.

### Electron microscopy

Negative staining electron microscopy (using 2% uranyl acetate) was done as described [38]. Rapid freezing/vitrification sample grid preparation for cryo-electron microscopy under oxygen-rich conditions (25% oxygen/75% nitrogen) was done as published [22]. Negatively stained specimens were studied with a FEI Tecnai 12 transmission electron microscope at an accelerating voltage of 120 kV. Images were taken with a 1392 × 1040 SIS Megaview camera. Cryo-electron microscopy was done with a FEI Polara transmission electron microscope, operating at an accelerating voltage of 300 kV and 59,000 × instrumental magnification. The negatives (Kodak SO 163 film) were developed for 12 minutes in full-strength Kodak D19 developer.

### Image processing

76 cryoEM micrographs of *Leptoxis carinata *hemocyanin (LcH) were digitised using a PRIMESCAN rotating drum scanner with a sampling size of 1-Å at the specimen level. The contrast transfer function (CTF) for each image was determined using the program CTFFIND3 [39]. 850 particles were manually picked with the module BOXER from the EMAN software package 1.7 [40], and extracted into 736 × 736 pixel boxes. The cut-out particles were corrected for the phase reversals of the CTF in IMAGIC [41], and, prior to band pass filtering and normalization, 2 × 2 averaged to give a final sampling of 2-Å/pixel.

Image processing was performed mainly with the IMAGIC software package. In case of *Leptoxis carinata *mega-hemocyanin, initial centring, classification and subsequent refinement of the class averages was done as described [22]. The initial map was calculated from class averages by angular reconstitution with imposed D5 symmetry and then refined using projection matching with an initial angular increment of 5° until there was no significant change in Euler angle assignments between successive refinement rounds. Subsequent refinement rounds were performed with decreasing angular steps (4°, 3°, 2° and 1°) until no further improvement could be detected. The final map was obtained from 627 superior particles, representing exclusively side-views of the tridecamer. The resolution of the final reconstruction was 13-Å according to the 1/2-bit criterion of the Fourier shell correlation [42].

In case of the KLH2 tridecamer, 97 negative staining EM micrographs were digitised with a sampling size of 2-Å and particles were manually picked within 368 × 368 boxes. A total of 110 single particles representing side views were manually collected. The final 30-Å (FSC_1/2-bit_) density map was calculated from 29 class-averages by angular reconstitution with imposed C5 symmetry.

### Rigid-body fitting and visualization

Automated rigid-body fitting of the molecular model of the KLH1 decamer [2] into the 30-Å cryoEM structure of the KLH2 tridecamer and the LcH tridecamer, respectively, was done in UCSF CHIMERA-1.3 [43], using the tool "fit in map". This was straightforward even in the 30-Å structure, because the right-handed helical wall features and the major elements of the collar complexes were clearly defined. The tool "color zone/split map" of the CHIMERA software was applied for segmentation of the maps; this procedure is based on the fitting. The molecular graphics images were also produced using CHIMERA.

### Oxygen binding curves

Continuous oxygen binding equilibrium curves for determination of oxygen affinity (p50) and cooperativity (Hill coefficient) of hemocyanin were recorded by the fluorimetric polarographic method [44]. The change in fluorescence as indicator of the oxygen saturation level was measured with a standard fluorimeter (Hitachi F4500, Binninger Analytic, Germany) at an emission wavelength of 338 nm (excitation 280 nm) while the oxygen concentration was determined simultaneously with an oxygen electrode (Microelectrodes Inc., Bedford, USA) equipped with a locally made amplifier. This electrode allowed determination of oxygen partial pressure to a precision of *ca. *1 mmHg. All experiments were performed at 20 ± 0.05°C at a protein concentration of 0.2 mg/ml in stabilizing buffer.

## Abbreviations

CTF: contrast transfer function; FSC: Fourier shell correlation; FU: functional unit; KLH: keyhole limpet hemocyanin (from *Megathura crenulata*); LcH: *Leptoxis carinata *hemocyanin; MtH: *Melanoides tuberculata *hemocyanin; PAGE: polyacrylamide gel electrophoresis; TpH: *Terebralia palustris *hemocyanin

## Competing interests

The authors declare that they have no competing interests.

## Authors' contributions

MGH, EES and BL provided *Leptoxis *and information on cerithioid snails. NH collected *Terebralia*. BL initiated the first electron microscopical images leading to the detection of mega-hemocyanin. BL, WG, CG, FD, NH and JM designed experiments and interpreted data. WG, CG, FD and NH performed the experiments. JM coordinated the experiments, produced the graphics and wrote the manuscript. All authors commented and approved the manuscript.
